# Editorial: Functional Imaging of Inflammation and Infection

**DOI:** 10.3389/fmed.2022.925635

**Published:** 2022-05-12

**Authors:** Ismaheel O. Lawal, Olivier Gheysens, Mike M. Sathekge, Andor W. J. M. Glaudemans

**Affiliations:** ^1^Department of Radiology and Imaging Sciences, Emory University, Atlanta, GA, United States; ^2^Department of Nuclear Medicine, University of Pretoria, Pretoria, South Africa; ^3^Department of Nuclear Medicine, Cliniques Universitaires Saint-Luc and Institute of Clinical and Experimental Research (IREC), Université Catholique de Louvain (UCLouvain), Brussels, Belgium; ^4^Nuclear Medicine Research Infrastructure (NuMeRI), Steve Biko Academic Hospital, Pretoria, South Africa; ^5^Medical Imaging Center, Department of Nuclear Medicine and Molecular Imaging, University of Groningen, University Medical Center Groningen, Groningen, Netherlands

**Keywords:** tuberculosis, myocarditis, idiopathic inflammatory myopathies, thyroiditis, FDG, [^68^Ga]Ga-NODAGA-RGD, [^68^Ga]Ga-FAPI

Inflammation is a host immune response to harmful stimuli, infectious or non-infectious, and is, therefore, a vital defense mechanism. Inflammation is thought to be the basis for many diseases, including oncology, and the effect transmissible infection could have on daily life has become clear to everyone in the past years. The importance arises from their associated morbidity and mortality as well as their socio-economic impact on health care. While the scourge of old infectious diseases such as tuberculosis continues to plague the human population, new ones are emerging, as exemplified by the still raging coronavirus disease (COVID-19) pandemic. To address the clinical and economic burden of inflammation and infection, prompt and accurate diagnosis of infection vs. sterile inflammation, timely treatment response determination, and the ability to prognosticate disease outcomes are essential. Diagnosis and treatment response assessment of infection and inflammation usually relies on clinical findings, measurement of circulating biomarkers, and histological or microbiological results. However, clinical manifestations are often non-specific, biopsy is invasive and fraught with sampling error, and serum biomarkers may also be non-specific and may not reflect the actual disease burden. Imaging, therefore, plays a vital role in the diagnosis and management of inflammation and infection. Morphologic imaging with computed tomography (CT) and magnetic resonance imaging (MRI) are commonly used imaging modalities in clinical practice. The distortion in tissue architecture induced by diseases and detectable by morphologic imaging techniques develops and resolves slowly, making them less ideal for prompt diagnosis and early response assessment.

This Research Topic, therefore, focuses on functional/molecular imaging of inflammation and infection to showcase the accuracy of whole-body imaging techniques that interphase functional imaging, especially with positron emission tomography (PET) in the diagnostic work-up and management of inflammation and infection.

[^18^F]-Fluorodeoxyglucose (FDG) is the PET radiopharmaceutical with the most robust evidence in inflammation and infection imaging. The impact of [^18^F]-FDG-PET/CT in the management of tuberculosis has been widely reported, even more in the assessment of treatment response, given the heterogeneity of the lesions. The largest burden of tuberculosis occurs in developing countries with limited access to functional imaging modalities. CT is a more widely available imaging modality in this region. In their original article, Lawal et al. showed a good correlation between residual metabolic activity in the lungs of patients who completed a standard course of anti-tuberculous therapy (a risk factor for disease relapse) and CT features of active pulmonary tuberculosis. As consequence, in regions where [^18^F]-FDG-PET/CT is not available, CT may be used as a valuable alternative for tuberculosis response assessment, especially in complicated cases where the standard of care procedures such as repeat sputum microscopy and culture becomes non-reliable.

The host inflammatory response to tuberculosis results in the formation of granuloma. In their review, More et al. presented an updated appraisal of the different molecular imaging targets that have been explored to image tuberculosis. The authors made a case for targeting neutrophils within the tuberculous granuloma due to the association between neutrophilic invasion of granuloma and poorer treatment outcomes.

Functional imaging with PET has the added advantage of standardized uptake quantification, which may help to quantify the extent of disease, evaluate response to treatment, and predict disease outcome. Idiopathic inflammatory myopathies (IIM) are a group of diseases characterized by muscle inflammation. The chronic disease course and the waxing and waning characteristics of IIM make [^18^F]-FDG-PET/CT a helpful imaging modality for lesion detection, quantifying the whole-body burden of disease, and evaluating response to anti-inflammatory therapy. Yildiz et al. provided a comprehensive review of published original studies evaluating the role of [^18^F]-FDG PET/CT -in IIM. [^18^F]-FDG-PET/CT showed a good overall diagnostic performance in evaluating disease activity using both visual qualitative and standardized uptake value (SUV)-based semi-quantitative metrics. In addition to detecting and quantifying disease extent, [^18^F]-FDG-PET/CT has the added value of detecting co-morbid conditions such as occult malignancy and inflammatory lung disease prevalent among patients with IIM and whose presence contributes to morbidity and mortality.

Despite the widespread application of [^18^F]-FDG-PET/CT in inflammatory and infectious diseases and a large body of evidence supporting its clinical use, the imaging modality has got some important limitations. FDG is an analog of glucose, which shows a high accumulation in organs with high basal glucose utilization or handling, such as the brain, heart, and the urinary system. The high radiotracer uptake in these organs may hamper the application of [^18^F]-FDG-PET/CT to assess diseases involving these regions. Therefore, there is a need to develop radiotracers without physiologic uptake in these organs. The development of one such tracer was addressed by the study of Jahandideh et al., who evaluated the feasibility to detect myocardial inflammation in a rat model of autoimmune myocarditis using [^68^Ga]Ga-NODAGA-RGD. This is a radiotracer that targets α_v_β_3_ integrin which is highly expressed in angiogenesis observed in inflammation. Unlike [^18^F]-FDG, [^68^Ga]Ga-NODAGA-RGD has minimal physiological myocardial uptake making it a suitable radiotracer for evaluating myocardial inflammation and infection. The authors showed significantly increased [^68^Ga]Ga-NODAGA-RGD uptake in the inflamed myocardium compared with non-inflamed myocardium, with the area of uptake corresponding to myocardial regions with myocardial necrosis and infiltration by inflammatory cells.

Fibroblast activating protein is highly expressed by activated fibroblasts seen in inflammation, infection, and tumors. Fibroblast activating protein inhibitors (FAPI) are small molecular targets of fibroblast-expressed FAP, which have been radiolabeled with Gallium-68 for PET imaging of various diseases. Being a fairly new radiotracer in clinical use, much remains to be known about the clinical significance of incidental uptake of radiolabeled FAPI in different organs. For the first time, Liu et al. explored the clinical significance of diffuse thyroid uptake of [^68^Ga]Ga-FAPI on PET/CT imaging obtained for different clinical indications. Diffuse thyroid uptake of [^68^Ga]Ga-FAPI was observed in 39 of 815 patients who underwent [^68^Ga]Ga-FAPI PET/CT scans for oncological reasons. The causes of diffuse tracer uptake in the thyroid of these patients were attributed to Hashimoto thyroiditis (most common) and Graves' disease.

This Research Topic presents a series of interesting articles showcasing the current clinical applications of functional imaging of inflammation and infection and the diverse targets that can be explored for future improvements.

## Author Contributions

IL: writing of the initial draft. OG, MS, and AG: revision and substantial intellectual contribution. All authors contributed to the article and approved the submitted version.

## Conflict of Interest

The authors declare that the research was conducted in the absence of any commercial or financial relationships that could be construed as a potential conflict of interest.

## Publisher's Note

All claims expressed in this article are solely those of the authors and do not necessarily represent those of their affiliated organizations, or those of the publisher, the editors and the reviewers. Any product that may be evaluated in this article, or claim that may be made by its manufacturer, is not guaranteed or endorsed by the publisher.

